# Spatial transcriptomics in the human adult ovary: insights into key signalling pathways during follicular atresia

**DOI:** 10.1093/humrep/deag051

**Published:** 2026-03-26

**Authors:** Hanne Vlieghe, Fu Wei, Hui Cheng, Tessa H R Stolk, Judith A Huirne, Norah M van Mello, Christiani A Amorim, Susana M Chuva de Sousa Lopes

**Affiliations:** Pôle de Recherche en Physiopathologie de la Reproduction, Institut de Recherche Expérimentale et Clinique, Université Catholique de Louvain, Brussels, Belgium; Department of Anatomy and Embryology, Leiden University Medical Center, Leiden, The Netherlands; The Novo Nordisk Foundation Center for Stem Cell Medicine (reNEW), Leiden University Medical Center, Leiden, The Netherlands; Department of Anatomy and Embryology, Leiden University Medical Center, Leiden, The Netherlands; The Novo Nordisk Foundation Center for Stem Cell Medicine (reNEW), Leiden University Medical Center, Leiden, The Netherlands; Department of Obstetrics and Gyneacology, Amsterdam University Medical Center, Amsterdam, The Netherlands; Centre of Expertise on Gender Dysphoria, Amsterdam UMC, Amsterdam, The Netherlands; Amsterdam Reproduction and Development Research Institute, Amsterdam, The Netherlands; Department of Obstetrics and Gyneacology, Amsterdam University Medical Center, Amsterdam, The Netherlands; Centre of Expertise on Gender Dysphoria, Amsterdam UMC, Amsterdam, The Netherlands; Amsterdam Reproduction and Development Research Institute, Amsterdam, The Netherlands; Department of Obstetrics and Gyneacology, Amsterdam University Medical Center, Amsterdam, The Netherlands; Centre of Expertise on Gender Dysphoria, Amsterdam UMC, Amsterdam, The Netherlands; Amsterdam Reproduction and Development Research Institute, Amsterdam, The Netherlands; Pôle de Recherche en Physiopathologie de la Reproduction, Institut de Recherche Expérimentale et Clinique, Université Catholique de Louvain, Brussels, Belgium; Department of Anatomy and Embryology, Leiden University Medical Center, Leiden, The Netherlands; The Novo Nordisk Foundation Center for Stem Cell Medicine (reNEW), Leiden University Medical Center, Leiden, The Netherlands; Ghent-Fertility and Stem Cell Team (G-FAST), Department of Reproductive Medicine, Ghent University Hospital, Ghent, Belgium

**Keywords:** human, ovary, adult, follicular atresia, folliculogenesis, spatial transcriptomics, steroidogenesis, signalling pathways

## Abstract

**STUDY QUESTION:**

Are key signalling pathways WNT, TGFβ/BMP, NOTCH, and HH involved in follicular atresia in the human adult ovary?

**SUMMARY ANSWER:**

In this study, we used spatial transcriptomics to investigate the progression of follicular atresia, focusing on genes of interest associated with steroidogenesis and key signalling pathways WNT, TGFβ/BMP, NOTCH, and HH.

**WHAT IS KNOWN ALREADY:**

While extensive research has focused on the mechanisms driving follicular growth, much less is known about the process of follicular atresia, despite its relevance for ovarian aging and reproductive longevity. Follicular atresia is characterized by complex molecular and cellular changes, that lead to the degeneration of granulosa and theca cells.

**STUDY DESIGN, SIZE, DURATION:**

Spatial transcriptomics was conducted on 16 regions of human ovarian tissue from different donors (N = 6) containing 21 small antral follicles (diameter 0.5–4 mm) healthy and at different stages of atresia.

**PARTICIPANTS/MATERIALS, SETTING, METHODS:**

We selected 80 genes to facilitate cell type identification in the ovary and to investigate the key signalling pathways WNT, TGFβ/BMP, NOTCH, and HH. Haematoxylin and eosin staining was used to manually select different follicle types for spatial transcriptomics. The Molecular Cartography platform (Resolve BioSciences) multiplexing single-molecule fluorescence *in situ* hybridization on cryo-sections was used for spatial transcriptomics. The cell segmentation masks were obtained from Resolve BioSciences and transcripts for each gene were assigned to individual cells based on the segmentation mask. Downstream visualization and quantification were performed using AnnData and Python.

**MAIN RESULTS AND THE ROLE OF CHANCE:**

By comparing the molecular signature of cell types present in healthy small antral follicles to those observed during the progression of atresia, we revealed a profound cellular and molecular shift. Key signalling pathways exhibited a general downregulation in granulosa cells, whereas expression in internal theca cells increased transiently at the onset of atresia, in line with ongoing cellular degeneration and follicular remodelling.

**LARGE SCALE DATA:**

N/A.

**LIMITATIONS, REASONS FOR CAUTION:**

This study was conducted on ovarian tissue from transmasculine donors. We cannot exclude that testosterone therapy impacts follicular dynamics.

**WIDER IMPLICATIONS OF THE FINDINGS:**

Our study provides novel insights into the spatial and molecular mechanisms of follicular atresia, contributing to a deeper understanding of the ovarian biology in humans.

**STUDY FUNDING/COMPETING INTEREST(S):**

This study was supported by the Fonds National de la Recherche Scientifique de Belgique (T.0004.20 to H.V. and C.A.A.), the Novo Nordisk Foundation (NNF21CC0073729, reNEW to F.W., H.C., and S.M.C.S.L.), the Dutch Organization for Health Research and Development (ZonMW PSIDER-2021-10250022120001 to S.M.C.S.L.), and the China Scholarship Council (CSC 202008450034 to F.W., CSC 202008320362 to H.C.). The authors have no conflicts of interest to declare.

## Introduction

As the primary functional unit of the adult ovary, the ovarian follicle is essential for both oocyte maturation and for hormone synthesis, playing a central role in female fertility (Gougeon, 2010). The pool of ovarian follicles is established before birth and during puberty, a cyclic process begins wherein a small number of follicles mature during each menstrual cycle. Of these follicles, typically one becomes dominant and will reach ovulation each menstrual cycle, while the others degenerate through atresia (McGee and Hsueh, 2000; Vaskivuo and Tapanainen, 2003). During follicular growth, the granulosa cells (GCs) proliferate and differentiate around the oocyte, providing essential support, while theca cells (TCs) provide structural integrity and contribute to hormone synthesis through steroidogenesis (Tajima *et al.*, 2006). The process of follicular growth and selection is governed by a complex network of signalling pathways, including phosphoinositide 3-kinase (PI3K)/AKT, WNT, NOTCH, HH, transforming growth factor-beta (TGFβ), and bone morphogenetic protein (BMP) pathways, which orchestrate the intricate cellular interactions in the follicular niche required for successful follicle maturation (Vo and Kawamura, 2021).

While extensive research has focused on the mechanisms driving follicular growth, less attention has been paid to the process of follicular atresia, despite its relevance for ovarian aging and reproductive longevity. Follicular atresia is characterized by complex molecular and cellular changes, such as reduced vascularization and the degeneration of GCs and TCs (Xie *et al.*, 2017). Atresia of small antral follicles seems to be prevented by high FSH levels (Baerwald *et al.*, 2012). After the dominant follicle is selected and FSH levels decrease, those small antral follicles start degenerating. This apoptotic process is hormone-controlled and starts with the loss of structural organization and GC loss. Furthermore, during this process, autophagosomes are observed and seem to play a role (Baerwald *et al.*, 2012). Recently, we have established a classification system for atresia in human small antral follicles, defining different atresia stages based on morphological and cellular criteria in comparisons to healthy follicles (Wei *et al.*, 2023). Healthy small antral follicles have well-organized layers of GCs, internal theca cells (in.TCs) and external theca cells (ex.TCs), but during the early signs of atresia (type 1) GCs start detaching from the follicular basement membrane and undergo apoptosis at the opposite pole of the cumulus. As atresia progresses (type 2), more pronounced structural changes emerge: GCs are gradually replaced by flat, fibroblast-like cells and the follicular basement membrane loses its integrity as infiltration by macrophages begins. In the last stage of atresia (type 3), GCs are entirely absent, the antral cavity becomes filled with fibroblast-like cells (ac.FIB), and the oocyte degenerates.

The specific effects of atresia on critical signalling pathways and somatic cell populations remain poorly understood. However, multiple signalling pathways are critically involved in the regulation of follicular growth and atresia. WNT/β-catenin signalling supports GC viability and steroidogenesis, and its disruption in mice is associated with increased follicular atresia (Fan *et al.*, 2010; Hernandez Gifford, 2015). TGFβ/BMP signalling, particularly mediated by oocyte-derived BMP15 and GDF9, suppresses GC apoptosis and maintains follicular growth, whereas inhibition of this pathway accelerates atresia (Yan *et al.*, 2001; Chang *et al.*, 2014). NOTCH signalling preserves GC proliferation and survival, and its blockade promotes apoptosis during follicular growth in mice (Vanorny *et al.*, 2014; Vanorny and Mayo, 2017). HH signalling further contributes to early follicular maintenance, with reduced activity linked to premature follicular loss in mice (Wijgerde *et al.*, 2005; Jiang *et al.*, 2019).

The advent of spatial transcriptomics offers a novel approach to understand transcriptional networks by enabling the analysis of gene expression in its spatial context within the human ovarian tissue. Unlike bulk or single-cell transcriptomics, spatial transcriptomics allows for the preservation of tissue architecture, facilitating a more comprehensive view of cellular interactions and gene regulation during follicular growth and atresia. Several recent studies have applied this technology to study mouse ovaries (Russ *et al.*, 2022; Mantri *et al.*, 2024; Wu *et al.*, 2024) and human ovaries (Jones *et al.*, 2024; Wu *et al.*, 2024). In mice, spatial transcriptomics has revealed significant differences in gene expression across cell populations in the ovaries from young versus old mice (Russ *et al.*, 2022) and between follicles of different maturation stages (Mantri *et al.*, 2024), providing valuable insights into the molecular dynamics of folliculogenesis. In humans, spatial transcriptomics highlighted differences in cell types between the ovarian cortex and medulla, and between GCs and TCs from a healthy small antral follicle, with findings validated by comparison to single-cell RNA sequencing (Jones *et al.*, 2024; Wu *et al.*, 2024). Despite these advances, the current studies mainly focus on healthy follicle development, leaving the molecular changes associated with follicular atresia largely unexplored.

In this study, we aimed to investigate the alterations in key signalling pathways WNT, TGFβ/BMP, NOTCH, and HH in somatic cell populations, such as GCs and TCs, during the progression of follicular atresia in small antral follicles (diameter 0.5–4 mm) in the adult human ovary. Using spatial transcriptomics, we provided novel insights underlying follicular remodelling, contributing to a better understanding of the human ovarian function and its implications for fertility.

## Materials and methods

### Ethical approval

This study was conducted in accordance with the Declaration of Helsinki. Signed informed consent was obtained from all tissue donors. The Medical Ethical Committee of the Leiden University Medical Center reviewed this study and issued a letter of no objection to the study (B18.029).

### Study population and tissue collection

This study used ovarian tissue samples from transmasculine donors of reproductive age (N = 6) between 18 and 26 years of age. Transmasculine donors received testosterone for at least 1 year and underwent gender-affirming oophorectomy at the Amsterdam University Medical Center. The ovaries were kept in ice-cold saline solution and transported to the laboratory at the Leiden University Medical Center within 1–2 h. Upon arrival, the ovaries were segmented in four to six pieces and embedded in optimal cutting temperature compound (O.C.T. Tissue-Tek, Sakura, Japan), snap-frozen in liquid nitrogen, and the cryo-blocks stored at −80°C.

### Histology and imaging

The cryo-blocks containing ovarian tissue pieces were sectioned (10 μm thickness) with a Leica CM3050 S Cryostat (Leica Instruments GmbH, Wetzlar, Germany), the tissue sections were placed on custom slides (Resolve BioSciences, Monheim am Rhein, Germany) and those slides were subsequently transported on dry ice to Resolve BioSciences. In addition, adjacent serial cryo-sections of the ovarian tissues were collected on StarFrost adhesive slides (3057-1, Walde mar Knittel, Braunschweig, Germany) to visualize tissue morphology using haematoxylin and eosin (H&E) following standard protocols. The H&E-stained slides were scanned with a Panoramic 250 digital scanner (3DHISTECH, Budapest, Hungary) and visualized through CaseViewer (v2.4.0) software (3DHISTECH, Budapest). Based on the morphological criteria established in our previous study (Wei *et al.*, 2023), different follicle types were identified using the H&E sections, and the follicle diameter was determined by measuring the longest axis.

### Spatial transcriptomics

Spatial transcriptomics was conducted on the human ovarian tissue by Resolve BioSciences using Molecular Cartography (protocol 3.0) (Resolve BioSciences, Monheim am Rhein, Germany) and their customized hybridization probes for 80 selected genes, representing the full set of probes used in this study. The probes used in this study were specifically selected to investigate the involvement of key signalling pathways WNT, TGFβ/BMP, NOTCH, and HH during follicular atresia in small antral follicles in the human adult ovary. This technology provides subcellular resolution as it uses single-molecule fluorescence *in situ* hybridization (smFISH). Sixteen selected regions of interest (2–25 mm^2^) were imaged using a Zeiss Celldiscoverer 7 equipped with a 50× Plan Apochromat water immersion objective (NA 1.2) and a 0.5× magnification changer (25× final magnification) at Resolve BioSciences, where the primary processing of the raw image data was conducted.

### Spatial transcriptomics data analysis

The visualization and exploration of the Molecular Cartography signals was initially performed using a web-based platform developed by Resolve BioSciences (https://my.resolvebiosciences.com). For downstream analysis, DAPI-stained nuclei images and transcript coordinates were uploaded to the platform, where cell segmentation was performed using a Bayesian-based method. This method used DAPI signal for nuclear segmentation and expanded outwards by 7.5 μm (default parameter) to define cellular boundaries. Subsequently, transcripts for each gene were assigned to individual cells based on the segmentation mask. The resulting cell segmentation mask and gene expression matrix were downloaded for further analysis using Python (v.3.10, accessed in the Netherlands).

An AnnData object was constructed using package Anndata (v0.10.9, accessed in the Netherlands) and subsequent data processing and analysis was performed using scanpy (v.1.11, accessed in the Netherlands). Function pp.filter_cells was used for quality control: cells with total counts <5 were removed and two genes (*MCAM* and *SYCP3*) not detected in the regions of interest (ROIs) were excluded. Gene expression (counts per gene per cell) was normalized (function: pp.normalize_total) by scaling to the target-sum [median of the total counts (sum of expression of the 80 genes of interest) per cell, calculated for the total cells present in the total ROIs]. In our data set, the target_sum was 104 counts/cell. This was followed by log-transformation (function: pp.log1p) to obtain Ln.Norm.Counts [ln (counts per gene per cell/target_sum + 1)] and for *Z*-score scaling (function: pp.scale).

For uniform manifold approximation and projection (UMAP) visualization, after using function pp.pca (n_comps = 50) and function pp.neighbors (n_neighbors = 15), cell clustering was performed using function tl.leiden (resolution = 0.4) and visualized using function tl.umap (min_dist = 0.5; spread = 1.0).

Differentially expressed genes (DEGs) for each identified cluster were calculated (function: tl.rank_genes_groups; method=‘wilcoxon’). DEGs were filtered based on adjusted *P*-value (*P*vals_adj <0.05) and percentage of non-zero counts per cluster (pct_nz_group >0.6) without applying a fold-change cutoff. The heatmaps were generated using function pl.matrixplot. Identified cell clusters and genes of interest were mapped to their spatial locations within the different ROIs using the Matplotlib (v.3.10.1, accessed in the Netherlands) based on the cell segmentation masks.

### Statistical analyses

We used ovarian material from 6 different transmasculine donors and selected 16 regions of interest that contained at least one follicle for spatial transcriptomics. In total, we obtained 251 421 cells from a total of 7 healthy small antral follicles, 4 type 1 atretic follicles, 4 type 2 atretic follicles, 5 type 3 atretic follicles. Statistical analysis to calculate differentially expressed genes was conducted using Wilcoxon test using adjusted *P*-value <0.05.

We further calculated the average gene expression per cell type of interest per follicle in the different types of follicles using customized code in Python (v.3.10). For this, the average of the normalized expression per gene per cell (Ln.Norm.Counts) for the total cells of each cell type of interest per follicle was calculated. Subsequently, the median expression per cell type for the different types of follicles was calculated and compared to healthy small antral follicles using the Mann–Whitney *U* test. Regarding the quantification, for category ‘granulosa cells’ (GC) we pooled cells from cluster 6 and 8 per follicle; for ‘internal theca cells’ (in.TC) we used cluster 3; for ‘external theca cells and stroma’ (ex.TC/ST) we pooled cells from cluster 0, 1, and 2 per follicle; for ac. FIB we pooled cells from cluster 7 and 9 per follicle; and all other cells per follicle were pooled in category ‘other’ (OTHER). *, *P* < 0.05; **, *P* < 0.01.

## Results

### Spatial transcriptomics reveals distinct clusters in healthy and atretic small antral follicles

**Figure 1.**
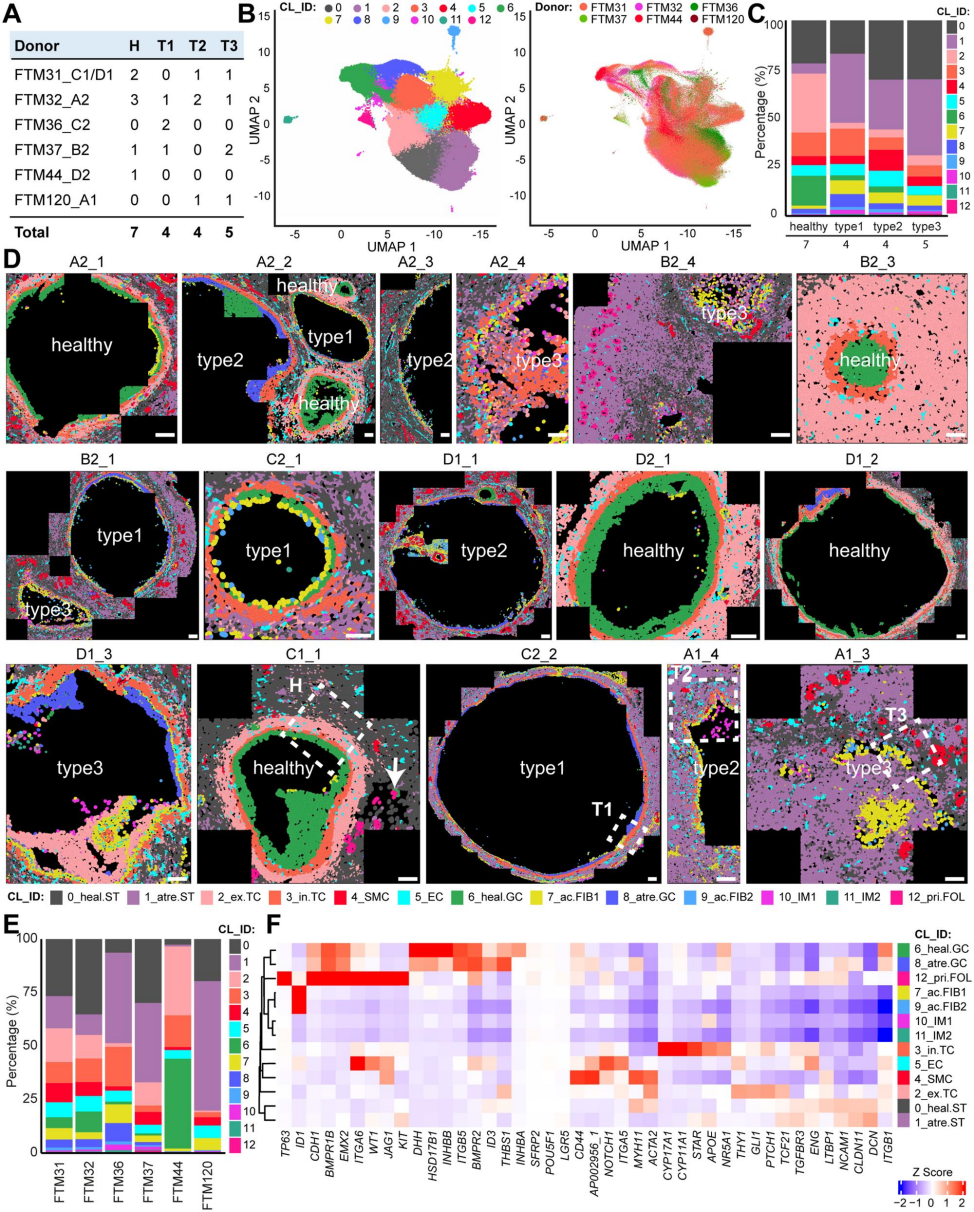
**Identification of cell types in healthy and atretic small antral follicles.** (**A**) List of individual donors (N = 6) and types of follicles (H, healthy; T1, atretic type 1; T2, atretic type 2; T3, atretic type 3) analysed per donor. (**B**) Uniform manifold approximation and projection (UMAP) plot showing segmented cells coloured by cluster (left panel) and by donor (right panel). (**C**) Distribution of cells per cluster per follicular type. (**D**) Spatial distribution of the identified cell clusters (CL_ID) projected in the segmentation mask of tissue sections of healthy (H), type 1 (T1), type 2 (T2), and type 3 (T3) atretic follicles in all regions of interest used in this study. White arrow (C1_1) points to a primordial follicle in the vicinity of a healthy small antral follicle. The areas depicted by the white dashed boxes, corresponding to H, T1, T2, and T3, are used magnified in all subsequent figures. Scale bar is 100 µm. (**E**) Distribution of cells per cluster per donor. (**F**) Heatmap showing the top 5 differentially expressed genes (DEGs) per cluster. Cluster identification (CL_ID): 0_heal.ST is healthy stroma; 1_atr.ST is atretic stroma; 2_ex.TC is external theca cells; 3_in.TC internal theca cells; 4_SMC is smooth muscle cells; 5_EC is endothelial cells; 6_heal.GC is healthy granulosa cells; 7_ac.FIB1 is fibroblast-like cells in antrum_1; 8_atr.GC is atretic granulosa cells; 9_ac.FIB2 is fibroblast-like cells in antrum_2; 10_IM1 is immune cells_1; 11_IM2 is immune cells_2; 12_pri.FOL is primordial/primary follicle.

We used spatial transcriptomics to investigate the expression pattern of 80 genes of interest, falling into two main categories: genes known to be expressed in specific cell types in the ovary to aid with cell identification and genes belonging to the key signalling pathways WNT, TGFβ/BMP, NOTCH, and HH (Supplementary Table S1) in ovarian tissue sections from 6 individual adult donors (N = 6) between 18 and 26 years of age (Supplementary Figs S1 and S2; Supplementary Table S2). From seven ovarian tissue sections, we selected 16 ROIs containing a total of 21 small antral follicles (diameter 0.5–4 mm) (Fig. 1A; Supplementary Figs S1 and S2; Supplementary Table S2). The selection of healthy follicles and follicles at different stages of atresia (type 1, with less organized GC layer, particularly pronounced at the basement membrane; type 2, with macrophages infiltrating the antral cavity; type 3, with filled antral cavity) was based on morphology using H&E staining (Supplementary Figs S1 and S2).

**Figure 2.**
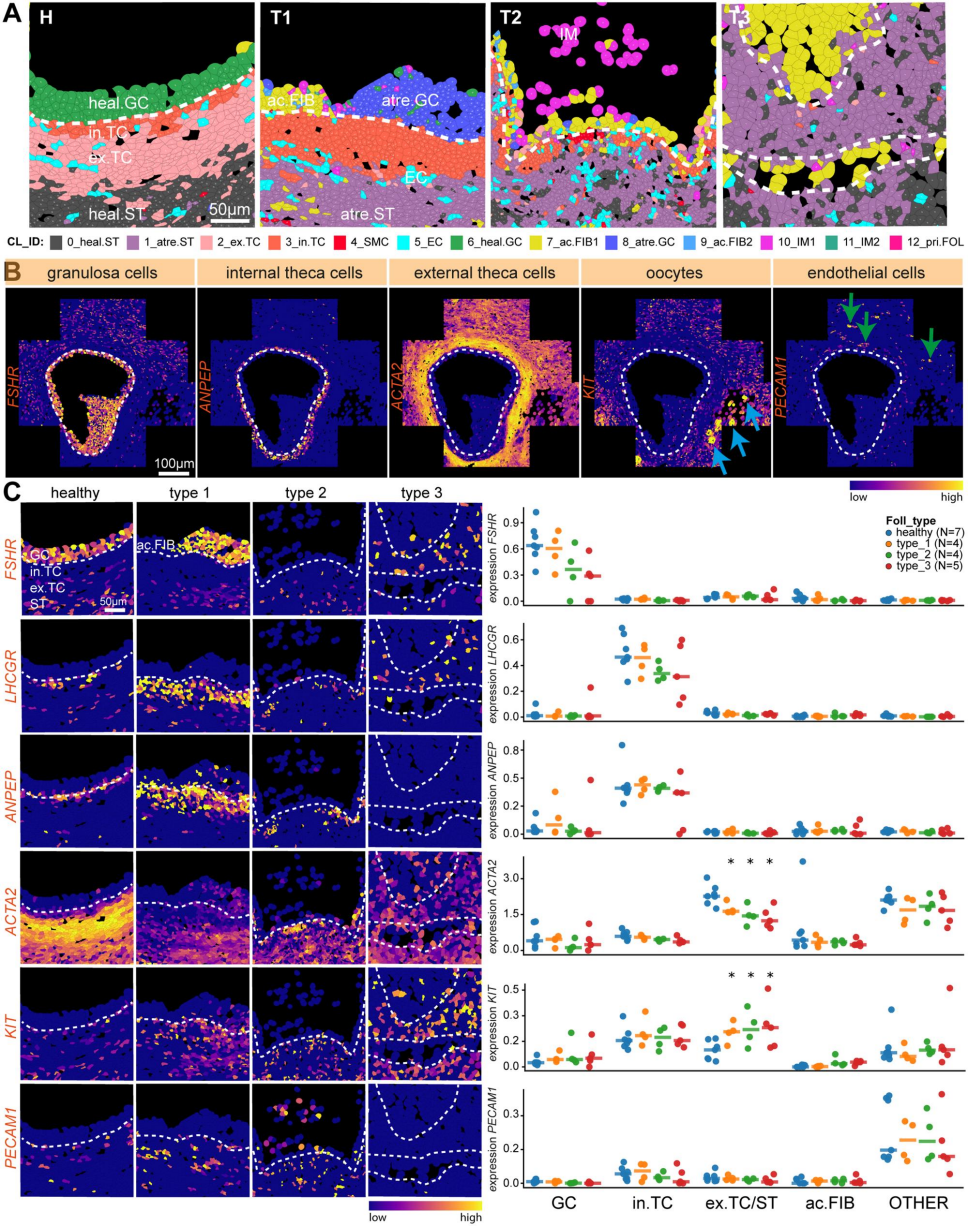
**Spatial cellular organization in healthy and atretic small antral follicles.** (**A**) Spatial distribution of the identified cell clusters (CL_ID) projected in the segmentation mask of tissue sections of healthy (H), type 1 (T1), type 2 (T2), and type 3 (T3) atretic follicles. Cluster identification (CL_ID): 0_heal.ST is healthy stroma; 1_atr.ST is atretic stroma; 2_ex.TC is external theca cells; 3_in.TC internal theca cells; 4_SMC is smooth muscle cells; 5_EC is endothelial cells; 6_heal.GC is healthy granulosa cells; 7_ac.FIB1 is fibroblast-like cells in antrum_1; 8_atr.GC is atretic granulosa cells; 9_ac.FIB2 is fibroblast-like cells in antrum_2; 10_IM1 is immune cells_1; 11_IM2 is immune cells_2; 12_pri.FOL is primordial/primary follicle. White dashed lines indicate the basement membrane of the small antral follicle. Scale bar is 50 µm. (**B**) Expression pattern of genes of interest projected in the segmentation mask of tissue sections of healthy small antral follicle (C1_1) to identify specific cells of interest (granulosa cells, internal theca cells, external theca cells, oocytes, and endothelial cells). Blue arrows point to *KIT*+ unilaminar follicles; green arrows point to *PECAM1*+ endothelial cells; white dashed line indicates the basement membrane of the small antral follicle. Scale bar is 100 µm. (**C**) Spatial distribution of gene expression projected in the segmentation mask of tissue sections of healthy, type 1, type 2, and type 3 atretic follicles in the same region as in **A.** Genes were selected to identify specific cells of interest (granulosa cells, internal theca cells, external theca cells, oocytes, and endothelial cells). White dashed lines indicate the basement membrane. Scale bar is 50 µm (left panels). Right panel shows the quantification of each gene per cell type of interest per follicle (GC, granulosa cells; in. TC, internal theca cells; ex. TC/ST, external theca cells and stromal cells; ac. FIB, fibroblast-like cells in antrum; and OTHER, all other cells per follicle). Each dot represents the average expression (Ln.Norm.Counts) per cell type per follicle and the horizontal stripe represents the median. The statistical test used to compare to healthy follicles was the Mann–Whitney *U* test; *P < 0.05.

Spatial transcriptomic analysis of these ROIs resulted in a dataset containing a total of 251 421 individually segmented cells, separated in 13 distinct clusters (CL), as visualized by UMAP (Fig. 1B), distributed in the different follicular types (Fig. 1C) and plotted on the segmented 16× ROIs (Fig. 1D). Except donor FTM44, that contributed with only one healthy follicle, all other donors contributed with both healthy and atretic small antral follicles (Fig. 1A, D, and E). We calculated the DEGs corresponding to each cluster (Supplementary Table S3), visualized the top 5 DEGs per cluster (ranked by log_fold_change) on a heatmap (Fig. 1F) and provided the *Z*-score for all analysed genes per cluster (Supplementary Fig. S3A). Mapping the identified clusters back onto the segmented cells in the ROIs provided a robust match between cell type identity (cluster annotation), considering the expected spatial distribution and nuclear morphology (based on DAPI) on the tissue section (Fig. 2A; Supplementary Fig. S3B). Moreover, we revealed distinct spatial patterns across follicle types, grossly separating healthy and atretic small antral follicles regarding the main follicular cell types (Figs 1D and 2A).

Healthy small antral follicles displayed healthy GCs (CL6) forming a cohesive layer around the antral cavity, while the theca layer was formed by in. TCs (CL3), ex. TCs (CL2) and contained endothelial cells (CL5). In the stromal compartment surrounding the healthy follicles (CL0), smooth muscle cells (CL4), and primordial/primary follicles (CL12) were also identified (Figs 1C and D and 2A). Note that the Resolve BioSciences platform was unable to separate oocytes and GCs in the few unilaminar follicles present near the small antral follicles adequately (Fig. 1D, follicle C1_1). Hence, those were not further analysed.

In type 1 atretic follicles, atretic GCs (CL8) dominated within the granulosa layer, indicating the onset of degeneration, where ac. FIB would gradually fill the antral cavity (CL7 and CL9). Interestingly, the stroma surrounding the type 1 follicles (and more advanced atretic follicles) also changed signature (CL1) compared to healthy stroma (CL0). From type 1 follicles onwards, the presumable immune cells, both inside (CL10) and outside (CL11) the follicle become prominent. The assignment of the immune cell identity was based on their specific cellular location in the antral cavity of type 2 follicles as previously reported (Wei *et al.*, 2023) and the absence of selected markers (Figs 1F and 2A; Supplementary Fig. S3A). In summary, this analysis provided a detailed spatial characterization of the cellular types associated with atresia in small antral follicles.

### Steroidogenesis and hormone receptors in small antral follicles during atresia

Next, we mapped several known specific makers into healthy follicles to validate the expected cell types in their location: GCs expressed *FSHR*, in. TCs expressed *ANPEP*, ex. TCs expressed high levels of *ACTA2*, oocytes expressed *KIT*, and endothelial cells expressed *PECAM1* (Fig. 2B and C). After quantification, we observed that *FSHR* remained expressed in GCs, and *LHCGR* and *ANPEP* remained expressed in the in. TCs during atresia, whereas the stroma upregulated *KIT* and downregulated *ACTA2* (Fig. 2C). The expression of *FSHR* and *LHCGR* confirmed that the small antral follicles analysed were gonadotropin-responsive.

We examined the expression of mesenchymal markers integrin beta 1 (*ITGB1*), *CD44*, *THY1*, and nerve growth factor receptor (*NGFR*) in the different clusters (Supplementary Fig. S3A) and across different stages of atresia (Supplementary Fig. S3B), but we were unable to identity a specific cluster of mesenchymal stem cells or enriched for those markers in small antral follicles (healthy and atretic). Interestingly, *ITGB1* and *CD44* were expressed in the immune cells present in the antral cavity in type 2 follicles (Supplementary Fig. S3B), in agreement with the presence of CD44+ macrophages in the antral cavity of atretic follicles in pig (Miyake *et al.*, 2006).

**Figure 3.**
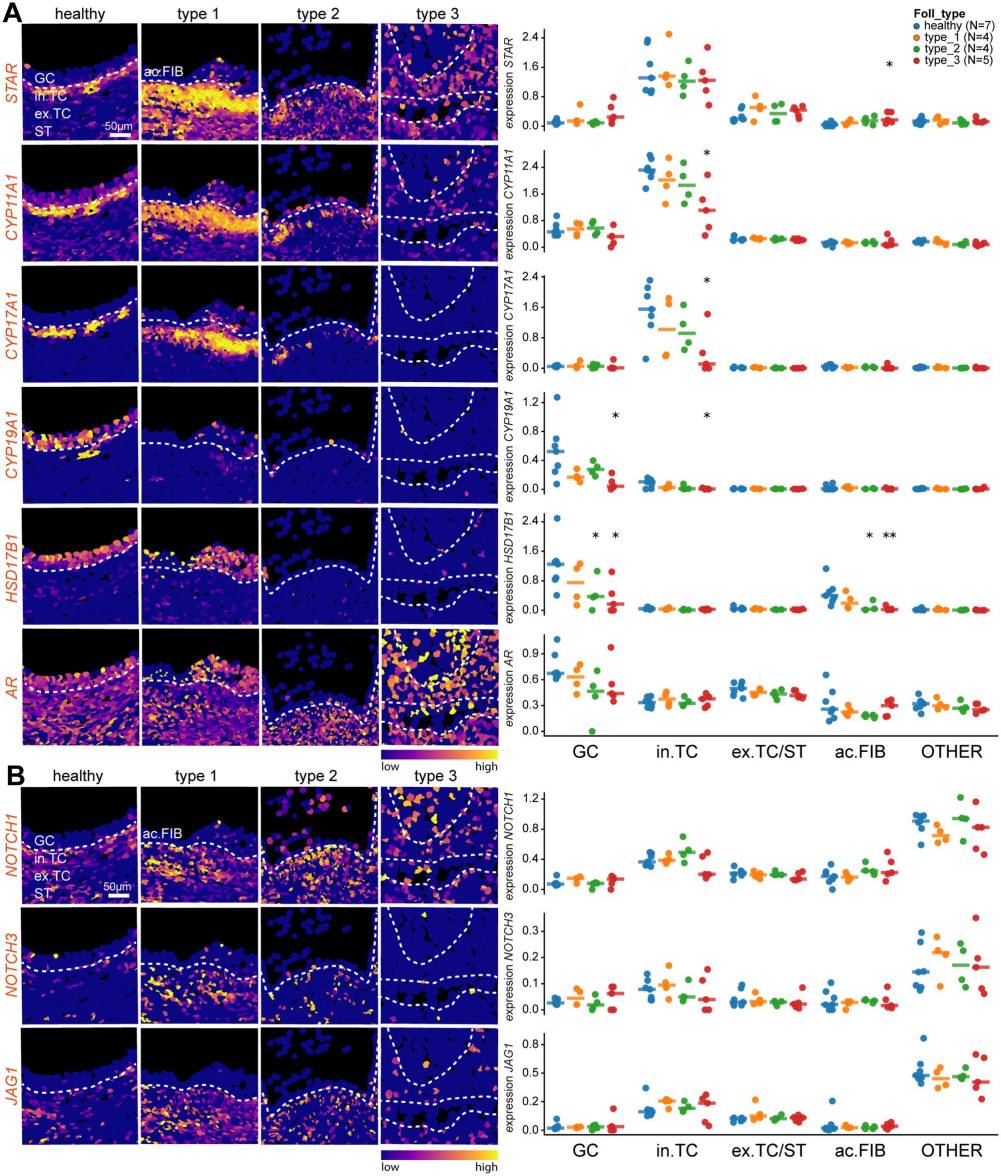
**Expression pattern of genes of interest associated with steroidogenesis and NOTCH signalling in healthy and atretic small antral follicles.** (**A**) Spatial distribution of gene expression of genes associated with steroidogenesis projected in the segmentation mask of tissue sections of healthy, type 1, type 2, and type 3 atretic follicles. White dashed lines indicate the basement membrane. Scale bar is 50 µm (left panels). Right panel shows the quantification of each gene per cell type of interest per follicle (GC, granulosa cells; in. TC, internal theca cells; ex. TC/ST, external theca cells and stromal cells; ac. FIB, fibroblast-like cells in antrum; and OTHER, all other cells per follicle). Each dot represents the average expression (Ln.Norm.Counts) per cell type per follicle and the horizontal stripe represents the median. The statistical test used to compare to healthy follicles was the Mann–Whitney *U* test; *P < 0.05; **P < 0.01. (**B**) Spatial distribution of gene expression of genes associated with NOTCH signalling projected in the segmentation mask of tissue sections of healthy, type 1, type 2, and type 3 atretic follicles. White dashed lines indicate the basement membrane. Scale bar is 50 µm (left panels). Right panel shows the quantification of each gene per cell type of interest per follicle (GC, granulosa cells; in. TC, internal theca cells; ex. TC/ST, external theca cells and stromal cells; ac. FIB, fibroblast-like cells in antrum; and OTHER, all other cells per follicle). Each dot represents the average expression (Ln.Norm.Counts) per cell type per follicle and the horizontal stripe represents the median. The statistical test used to compare to healthy follicles was the Mann–Whitney *U* test, but none of the comparisons was significantly different.

Steroidogenesis is a crucial process during folliculogenesis, that occurs in both GCs and in. TCs (Zheng *et al.*, 2023). In healthy antral follicles and type 1 atretic follicles, the expression of *STAR*, *CYP17A1*, and *CYP11A1*, coding for essential steroidogenic enzymes, was mainly observed in in. TCs (Fig. 3A). Further along the steroidogenesis pathway, *CYP19A1* and *HSD17B1*, both critical for oestrogen synthesis, were primarily expressed in healthy GCs and decreased significantly during atresia (Fig. 3A). Interestingly, *CYP19A1* and *HSD17B1* also showed downregulation in atretic in. TC and ac. FIB, respectively (Fig. 3A). Interestingly, *AR* was expressed in most cells in both healthy and atretic small antral follicles, with the exception of the immune cells (Fig. 3A). Together, our results suggest that the expression of androgens by the in. TCs may play a role in the onset of atresia.

### NOTCH signalling pathway in small antral follicles during atresia

The NOTCH signalling pathway is important during folliculogenesis (Vanorny and Mayo, 2017). Interestingly, *NOTCH1*, *NOTCH3*, and *JAG1* were not expressed in GCs, but showed some expression in in. TCs, and particular strong expression in endothelial cells (CL5) (Fig. 3B; Supplementary Fig. S3A). Moreover, *NOTCH1* was detected in the immune cells in the antral cavity of type 2 atretic follicles as well as in ac. FIB (CL7) in type 3 atretic follicles (Fig. 3B; Supplementary Fig. S3A), suggesting a direct involvement of this signalling pathway in follicular atresia.

### WNT signalling pathway in small antral follicles during atresia

**Figure 4.**
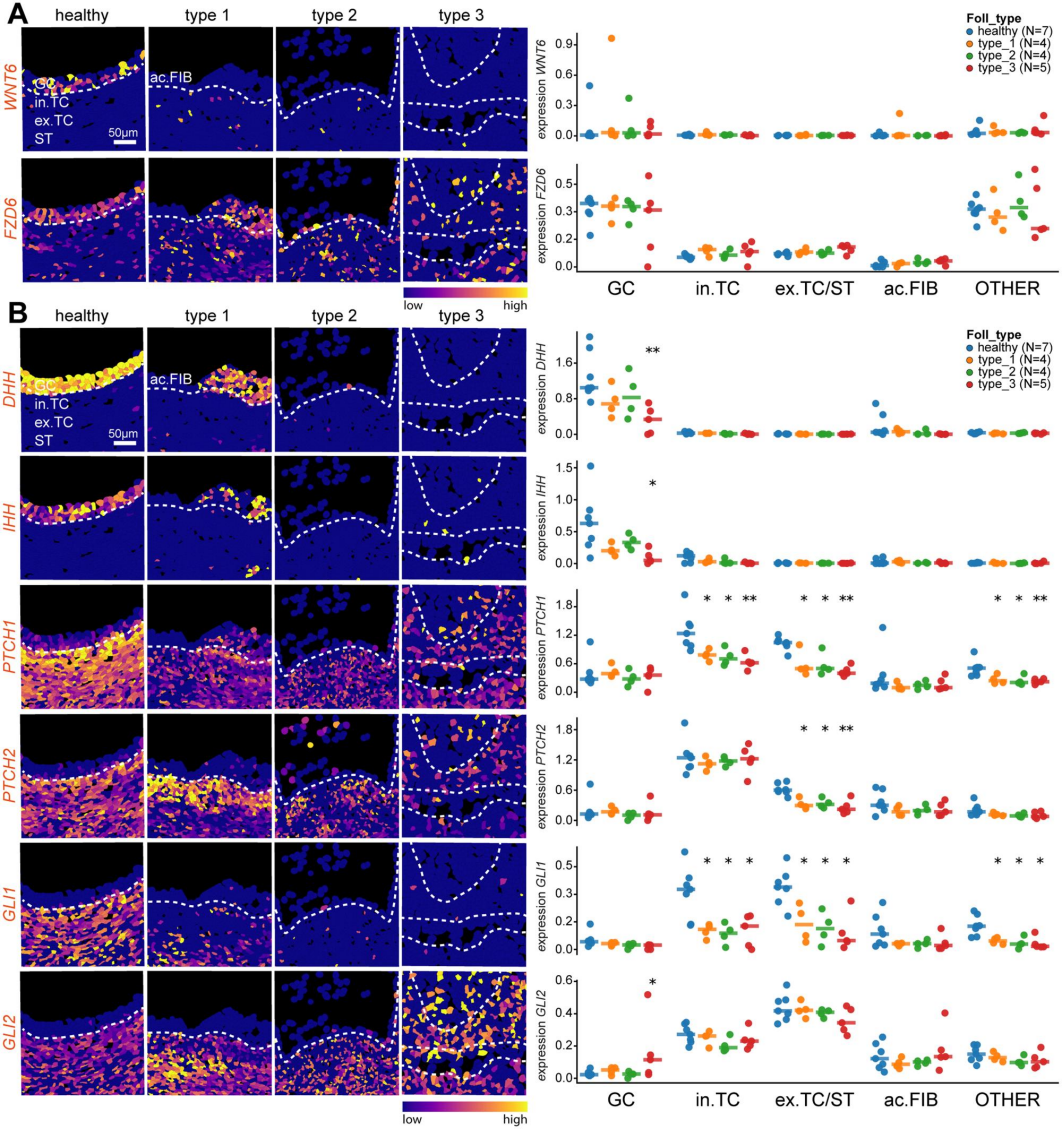
**Expression pattern of genes of interest associated with WNT and HH signalling in healthy and atretic small antral follicles.** (**A**) Spatial distribution of gene expression of genes associated with WNT signalling projected in the segmentation mask of tissue sections of healthy, type 1, type 2, and type 3 atretic follicles. White dashed lines indicate the basement membrane. Scale bar is 50 µm (left panels). Right panel shows the quantification of each gene per cell type of interest per follicle (GC, granulosa cells; in. TC, internal theca cells; ex. TC/ST, external theca cells and stromal cells; ac. FIB, fibroblast-like cells in antrum; and OTHER, all other cells per follicle). Each dot represents the average expression (Ln.Norm.Counts) per cell type per follicle and the horizontal stripe represents the median. The statistical test used to compare to healthy follicles was the Mann–Whitney *U* test, but none of the comparisons was significantly different. (**B**) Spatial distribution of gene expression of genes associated with HH signalling projected in the segmentation mask of tissue sections of healthy, type 1, type 2, and type 3 atretic follicles. White dashed lines indicate the basement membrane. Scale bar is 50 µm (left panels). Right panel shows the quantification of each gene per cell type of interest per follicle (GC, granulosa cells; in. TC, internal theca cells; ex. TC/ST, external theca cells and stromal cells; ac. FIB, fibroblast-like cells in antrum; and OTHER, all other cells per follicle). Each dot represents the average expression (Ln.Norm.Counts) per cell type per follicle and the horizontal stripe represents the median. The statistical test used to compare to healthy follicles was the Mann–Whitney *U* test; *P < 0.05; **P < 0.01.

*WNT6*, coding for a key ligand in the WNT signalling pathway, was specifically expressed in the GCs of a few small antral follicles (Fig. 4A). However, the expression of its receptor *FZD6* was prominent in the GCs of healthy and atretic follicles, and was also observed in the stromal compartment (Fig. 4A). Our results suggest that although WNT pathway may pay an important role in proliferation and maturation of GCs in small antral follicles in humans, it does not seem to play a prominent role during follicular atresia.

### HH signalling pathway in small antral follicles during atresia

Expression of the components of the HH signalling has been reported in the ovaries of mice (Wijgerde *et al.*, 2005). In mice adult ovaries, *Dhh* and *Hhh* were observed in GCs, whereas *Ptch1*, coding for receptor, and *Gli1*, coding for the downstream effector, were expressed in the TCs of antral follicles (Wijgerde *et al.*, 2005). In agreement, we observed a comparable expression pattern in healthy follicles in humans. *DHH* and *IHH* were highly expressed in the GCs of healthy and type 1 follicles, but decreased significantly during atresia. The HH pathway receptors *PTCH1* and *PTCH2*, and downstream effectors *GLI1* and *GLI2* were primarily expressed in TCs and stroma in healthy follicles (Fig. 4B). In type 1 follicles, *PTCH1* was downregulated in in. TCs; and both receptors and effectors were expressed in the stromal compartment suggesting that the HH could be activated during atresia.

### TGFβ/BMP signalling pathway in small antral follicles during atresia

**Figure 5.**
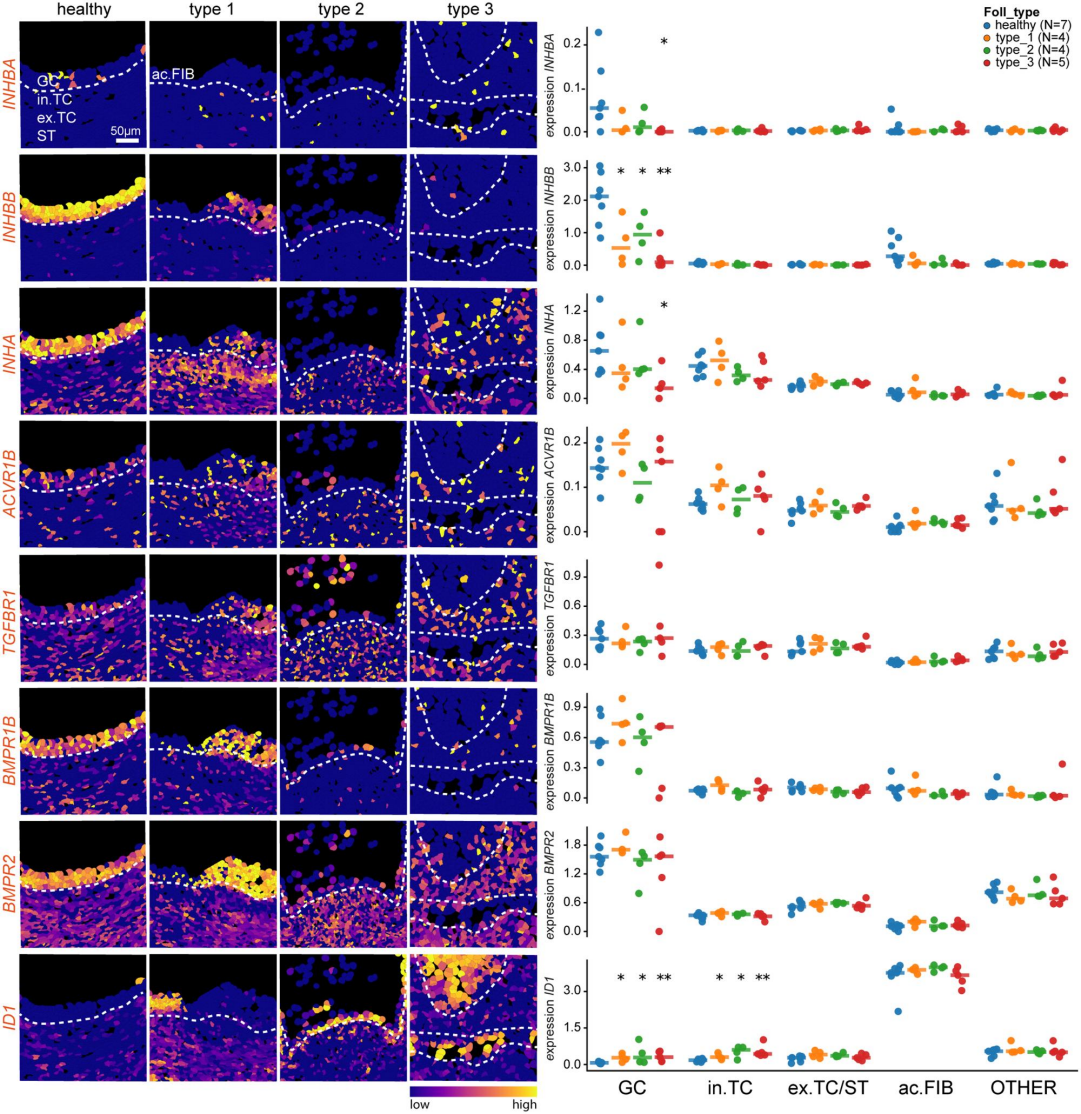
**Expression pattern of genes of interest associated with TGFβ/BMP signalling in healthy and atretic small antral follicles.** Spatial distribution of gene expression of genes associated with TGFβ/BMP signalling projected in the segmentation mask of tissue sections of healthy, type 1, type 2, and type 3 atretic follicles. White dashed lines indicate the basement membrane. Scale bar is 50 µm (left panels). Right panel shows the quantification of each gene per cell type of interest per follicle (GC, granulosa cells; in. TC, internal theca cells; ex. TC/ST, external theca cells and stromal cells; ac. FIB, fibroblast-like cells in antrum; and OTHER, all other cells per follicle). Each dot represents the average expression (Ln.Norm.Counts) per cell type per follicle and the horizontal stripe represents the median. The statistical test used to compare to healthy follicles was the Mann–Whitney *U* test; *P < 0.05; **P < 0.01.

The TGFβ/BMP signalling pathway plays a critical role in ovarian function, mediated by ligands such as Activins, including Activin A (homodimer of *INHBA*), Activin B (homodimer of *INHBB*), Activin AB (heterodimer of *INHBA* and *INHBB*) that stimulate production of FSH; and Inhibins, Inhibin A (heterodimer of *INHA* and *INHBA*) and Inhibin B (heterodimer of *INHA* and *INHBB*), that inhibit FSH secretion (Chand *et al.*, 2010). Mutations in *INHA* have been associated with premature ovarian failure (Chand *et al.*, 2010). We observed high expression of *INHA* and *INHBB* and low expression of *INHBA* in GCs of healthy follicles and to a lesser extent in GCs in type 1 follicles (Fig. 5), confirming that Inhibin B is produced by GCs of healthy small antral follicles as expected (Chand *et al.*, 2010). During atresia, we observed a significant downregulation of *INHBB* and *INHA* in GCs, but *INHA* remained in TCs in type 1 follicles and stroma (Fig. 5). The TGFβ type I receptors, *TGFBR1* (or *ALK5*) and *ACVR1B* (or *ALK4*) were expressed at low levels in all cell types, except by ac. FIB (Fig. 5), suggesting that TGFβ signalling can be activated in the ovary during remodelling.

Regarding the BMP signalling pathway, both BMP type I receptor *BMPR1B* (or *ALK6*) and BMP type II receptor *BMPR2* were highly expressed in GCs of healthy small antral follicles and during atresia, indicating that this pathway can be activated in GCs and confirming a potential role in atresia progression in human follicles as demonstrated in cattle (Gasperin *et al.*, 2014). *BMPR2* was expressed in most other cell types, except by ac. FIB (Fig. 5). Interestingly, *ID1*, a downstream gene of BMP signalling, was prominently expressed by the ac. FIB and lack expression of TGFβ/BMP type I receptors (Fig. 5), suggesting that *ID1* expression there may be independent of TGFβ/BMP signalling.

## Discussion

This study used spatial transcriptomics with a targeted set of genes to gain deeper insights into the involvement of key signalling pathways (WNT, TGFβ/BMP, NOTCH, and HH) in the adult human ovary during progression of atresia in small antral follicles. Our analysis demonstrated distinct changes in expression patterns that reflected the breakdown of the follicular structure in line with our previous classification of follicular atresia (Wei *et al.*, 2023).

Stromal cells are integral components of the ovarian microenvironment, providing both structural support and facilitating cellular communication that is essential for follicular health and function (Kinnear *et al.*, 2020; Grubliauskaite *et al.*, 2024). In our study, stromal cells surrounding the small antral follicles exhibited notable gene expression changes throughout the progression of follicular atresia, suggesting active involvement in the remodelling process.

Steroidogenesis plays a critical role in follicular development in humans (Zheng *et al.*, 2023). Here, we showed that steroidogenesis and in particular androgen production as well as responsiveness to luteinizing hormone by the in. TCs may play and important role during the onset of atresia. It remains unclear whether in. TCs shift identity towards ac. FIB, as previously suggested (Tajima *et al.*, 2007), perhaps dedifferentiating into less specialized state with limited steroidogenic activity (Erickson *et al.*, 1985). Future studies could investigate whether in. TCs undergo phenotypical changes, contributing to ovarian remodelling.

Many signalling pathways are known to be involved in follicular development in mammals and in particular in humans (Juengel and McNatty, 2005; Li *et al.*, 2021). Our results showed that ligands of the key signalling pathways WNT, TGFβ/BMP, and HH were expressed in GCs of healthy follicles, whereas receptors and effectors were mainly expressed in TCs and stroma, not only in healthy follicles, but also during follicular atresia, suggesting that follicular cells can activate these signalling pathways in the ovary during follicular remodelling. Interestingly, BMP receptors were highly expressed in the GCs from type 1 follicles in humans, as observed in cattle (Gasperin *et al.*, 2014). Our results underscore the complexity of atresia, revealing that it is not merely a passive degeneration but potentially an active process involving signalling adjustments that may aid in the maintenance of ovarian structure.

This study has several limitations. We cannot exclude that the size of the ROI influences the number of detected genes per segmented cell and the downstream differential expression analysis. To mitigate this, for quantification we have analysed cells from different follicles in different ROIs separately. Moreover, although the Resolve BioSciences platform was adequate to segment somatic cells (with regular size and nuclear DAPI), the platform was less suitable to segment the oocyte (with large size and low DAPI). Hence oocytes were not analysed further. Our selected gene panel did not contain immune cells markers and (presumable) immune cell clusters were classified based on cellular morphology and specific location in the follicle (Wei *et al.*, 2023). An important consideration in interpreting our findings is also the potential impact of testosterone therapy on follicular dynamics and cellular pathways in the ovarian tissue of transmasculine people. Transmasculine individuals may retain ovulatory function despite testosterone therapy (Asseler *et al.*, 2024), hence inter-individual differences in the suppression of the hypothalamic-pituitary-ovarian (HPO) axis could contribute to variability in small antral follicle dynamics and atresia. Although we have not observed significantly increased numbers of atretic follicles in ovarian tissue of transmasculine people (Wei *et al.*, 2023), it remains to be determined whether increased testosterone levels accelerate degeneration of small antral follicles. To exclude this, it would be important to validate our results in ovaries from other donor groups, although we recognize the difficulties in obtaining comparable samples from cis donors.

Concluding, using spatial transcriptomics for selected genes of interest using a platform that allows for single-cell spatial resolution revealed the dynamic distribution of ovarian cell types in small antral follicles during the progression of atresia in humans, an important aspect in the process of ongoing ovarian remodelling. These findings contribute to a deeper understanding of human ovarian biology and open new perspectives for therapeutic strategies and potential applications, by identifying candidates that could reduce follicular atresia, modulating ovarian ageing to improve reproductive longevity.

## Supplementary Material

deag051_Supplementary_Figure_S1

deag051_Supplementary_Figure_S2

deag051_Supplementary_Figure_S3

deag051_Supplementary_Table_S1

deag051_Supplementary_Table_S2

deag051_Supplementary_Table_S3

## Data Availability

The datasets generated during and/or analysed during the current study are available from the corresponding author on reasonable request. The code used in this study is available on GitHub (https://github.com/chuvalab/ova_resolve) and the original code is deposited at Zenodo (DOI 10.5281/zenodo.17669193).
