# Correction to: Spatial transcriptomics in the human adult ovary: insights into key signalling pathways during follicular atresia

**DOI:** 10.1093/humrep/deag080

**Published:** 2026-05-11

This is a correction to: Hanne Vlieghe, Fu Wei, Hui Cheng, Tessa H R Stolk, Judith A Huirne, Norah M van Mello, Christiani A Amorim, Susana M Chuva de Sousa Lopes, Spatial transcriptomics in the human adult ovary: insights into key signalling pathways during follicular atresia, *Human Reproduction*, 2026; https://doi.org/10.1093/humrep/deag051

This paper was published with supplementary files lacking from the **Supplementary Data** section. The files have now been supplied to this paper.

