# Supplementary material for: Spatial transcriptomics in the human adult ovary: insights into key signalling pathways during follicular atresia
Source: Hum Reprod. 2026 Mar 26;41(6):929–39. doi: 10.1093/humrep/deag051 (PMC13230497; doi:10.1093/humrep/deag051)
Supplement: deag051_Supplementary_Figure_S1 [file deag051_supplementary_figure_s1.pdf]

OXFORD
human reproduction
Human Reproduction, 2026, 1–1
https://doi.org/10.1093/humrep/deag051
Supplementary Data

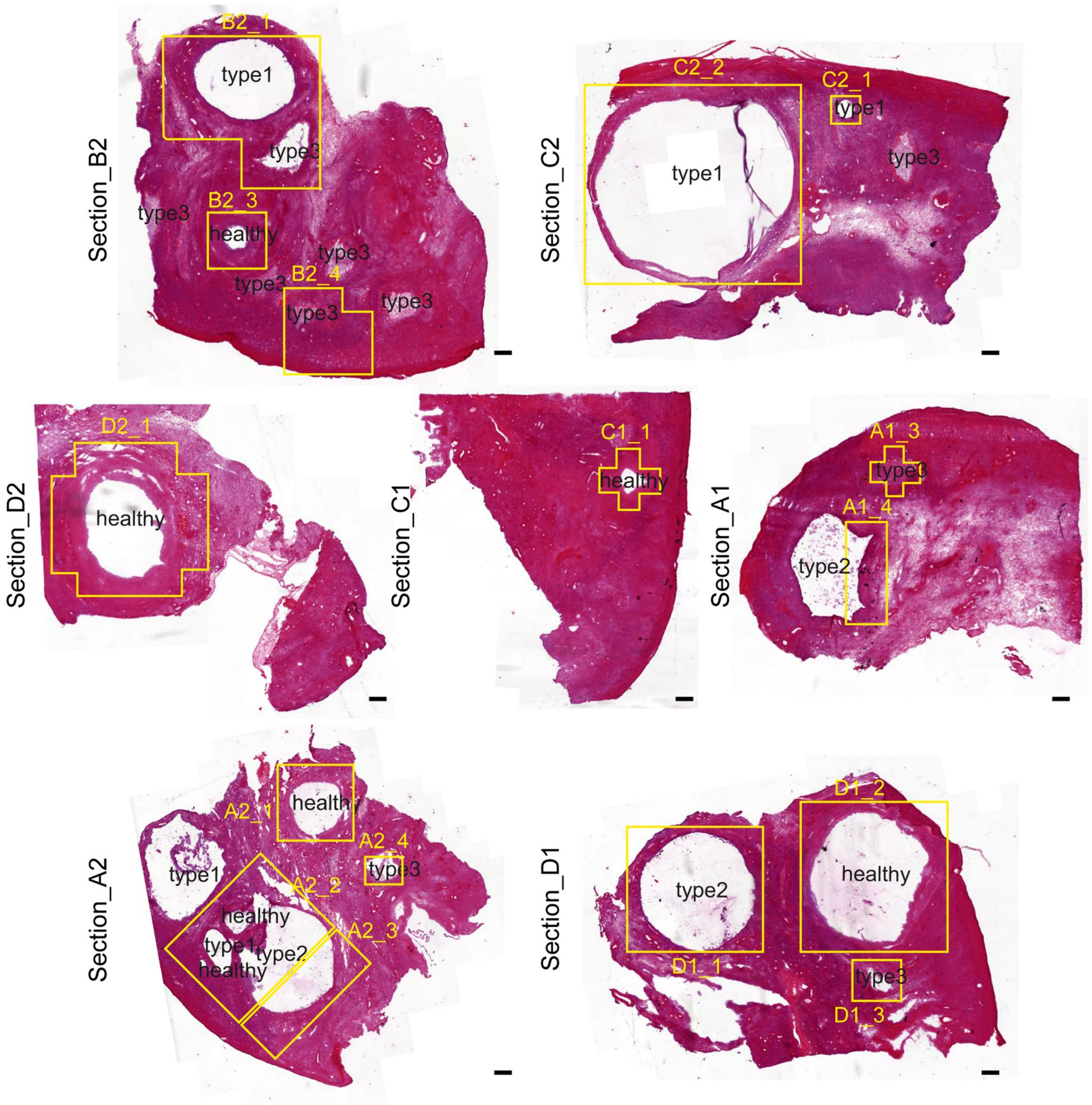

Supplementary Figure S1. **Overview of the ovarian sections used for spatial transcriptomics.** Hematoxylin and eosin (H&E) staining of the complete ovarian sections used for spatial transcriptomics. The selected regions of interest are depicted in yellow. The different types of follicles present in each section were classified as healthy, type 1, type 2, and type 3 atretic follicles according to morphological characteristics. Scale bars are 500 μm.
