# Supplementary material for: Spatial transcriptomics in the human adult ovary: insights into key signalling pathways during follicular atresia
Source: Hum Reprod. 2026 Mar 26;41(6):929–39. doi: 10.1093/humrep/deag051 (PMC13230497; doi:10.1093/humrep/deag051)
Supplement: deag051_Supplementary_Figure_S2 [file deag051_supplementary_figure_s2.pdf]

OXFORD
human reproduction
Human Reproduction, 2026, 1–1
https://doi.org/10.1093/humrep/deag051
Supplementary Data

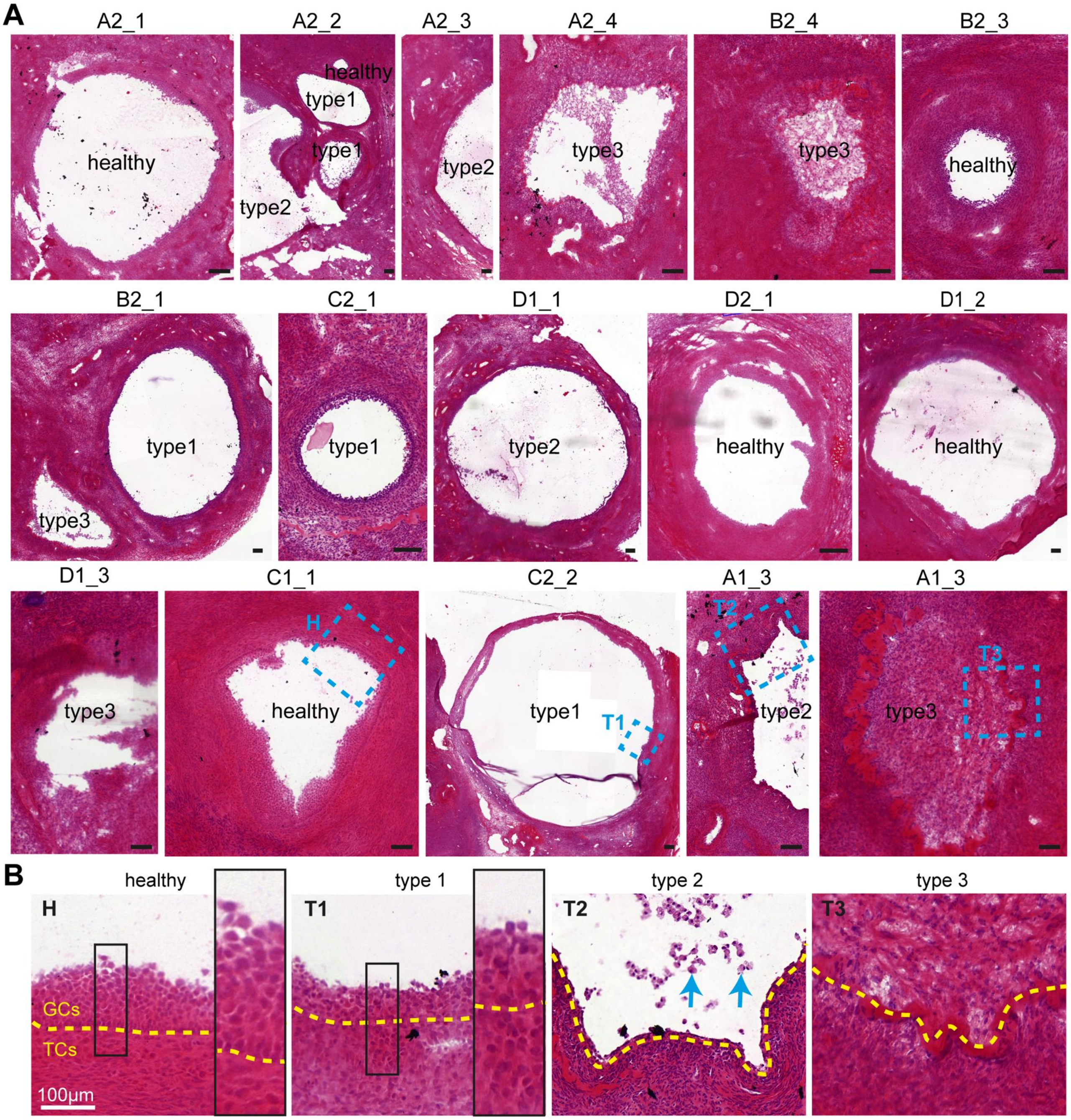

Supplementary Figure S2. Regions of interest (ROIs) selected for spatial transcriptomics. (**A**) Hematoxylin and eosin (H&E) staining of all the ovarian regions of interest used for spatial transcriptomics. The different types of follicles analysed were classified as healthy, type 1, type 2, and type 3 atretic follicles. Blue dashed boxes indicate regions magnified in (B). Scale bars are 100 µm. (**B**) H&E staining of healthy (H), type 1 (T1), type 2 (T2), and type 3 (T3) atretic follicles, illustrating distinct morphological features. Yellow dashed line indicates the basement membrane between granulosa cells (GCs) and theca cells (TCs). Blue arrows indicate macrophages in the antral cavity. For H and T1, a black box is shown magnified left to contrast the organization of the GC layer in H, in particular the tight arrangement of the GCs contacting the basement membrane; with the more disorganized GC layer in T1. Scale bar is 100 µm.
