# Supplementary material for: Spatial transcriptomics in the human adult ovary: insights into key signalling pathways during follicular atresia
Source: Hum Reprod. 2026 Mar 26;41(6):929–39. doi: 10.1093/humrep/deag051 (PMC13230497; doi:10.1093/humrep/deag051)
Supplement: deag051_Supplementary_Figure_S3 [file deag051_supplementary_figure_s3.pdf]

OXFORD
human reproduction
Human Reproduction, 2026, 1–1
https://doi.org/10.1093/humrep/deag051
Supplementary Data

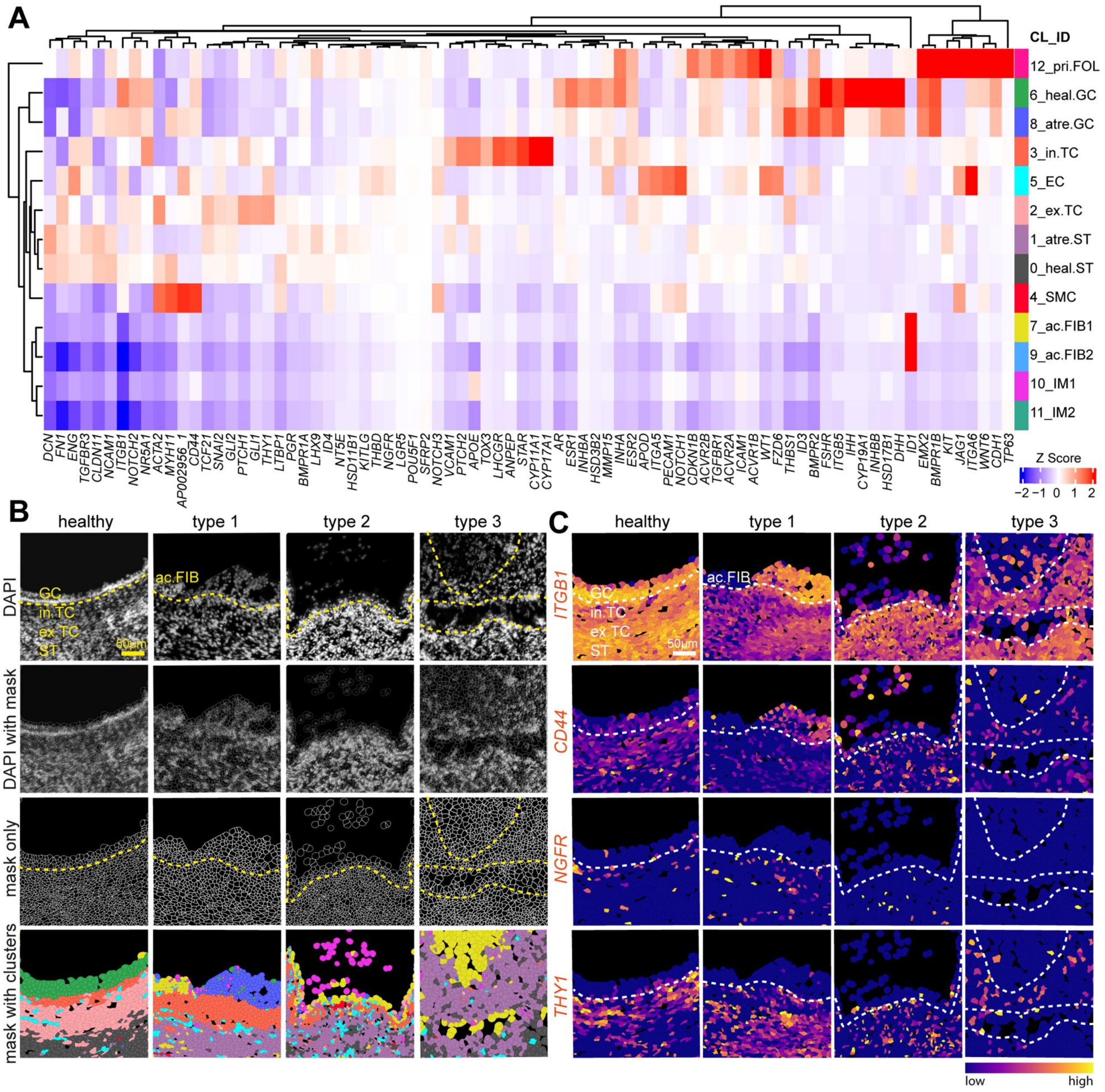

Supplementary Figure S3. Overview of gene expression levels per cluster, segmentation mask, and expression of genes of interest. (**A**) Heatmap showing Z-score of all genes analysed per cluster. Cluster identification (CL_ID): 0_heal.ST is healthy stroma; 1_atr.ST is atretic stroma; 2_ex.TC is external theca cells; 3_in.TC internal theca cells; 4_SMC is smooth muscle cells; 5_EC is endothelial cells; 6_heal.GC is healthy granulosa cells; 7_ac.FIB1 is fibroblast-like cells in antrum_1; 8_atr.GC is atretic granulosa cells; 9_ac.FIB2 is fibroblast-like cells in antrum_2; 10_IM1 is immune cells_1; 11_IM2 is immune cells_2; 12_pri.FOL is primordial/primary follicle. (**B**) Segmentation mask based on DAPI staining. Using the DAPI signal of the tissue sections (top row), a segmentation mask was generated automatically in the Resolve BioSciences platform (using 7.5μm as default parameter to expand outwards) to define cellular boundaries (middle rows). Subsequently, the generated segmentation mask was used to visualize assigned cell clusters (bottom row) and gene expression throughout the manuscript. Scale bar is 50 μm. (**C**) Spatial distribution of gene expression of genes of interest projected in the segmentation mask of tissue sections of healthy, type 1, type 2, and type 3 atretic follicles. White dashed lines indicate the basement membrane. Scale bar is 50 μm.
