# Supplementary material for: Spatial transcriptomics in the human adult ovary: insights into key signalling pathways during follicular atresia
Source: Hum Reprod. 2026 Mar 26;41(6):929–39. doi: 10.1093/humrep/deag051 (PMC13230497; doi:10.1093/humrep/deag051)
Supplement: deag051_Supplementary_Table_S1 [file deag051_supplementary_table_s1.pdf]

Human Reproduction, 2026, 1–1
https://doi.org/10.1093/humrep/deag051
Supplementary Data

Supplementary Table S1. List of selected genes used for spatial transcriptomics.

Gene	Note
ACTA2	Theca externa/smooth muscle cell
ACVR1B	TGFb/BMP signaling
ACVR2A	TGFb/BMP signaling
ACVR2B	TGFb/BMP signaling
ANPEP	Theca interna
AP002956.1	Smooth muscle cell
APOD	Steroidogenesis
APOE	Steroidogenesis
AR	Granulosa
BMPR1A	TGFb/BMP signaling
BMPR1B	TGFb/BMP signaling
BMPR2	TGFb/BMP signaling
CD44	Mesenchymal/macrophage
CDH1	Epithelial–mesenchymal transition
CDKN1B	Cell proliferation
CLDN11	Granulosa
CYP11A1	Theca interna/steroidogenesis
CYP17A1	Theca interna/steroidogenesis
CYP19A1	Granulosa/steroidogenesis
DCN	Fibroblast
DHH	Hedgehog signaling
EMX2	Granulosa
ENG	Mesenchymal
ESR1	Granulosa/steroidogenesis
ESR2	Granulosa/steroidogenesis
FN1	Fibroblast
FSHR	Granulosa
FZD6	WNT signalling
GLI1	Hedgehog signaling
GLI2	Hedgehog signaling
HSD3B2	Steroidogenesis
HSD11B1	Steroidogenesis
HSD17B1	Granulosa/steroidogenesis
ICAM1	Granulosa
ID1	TGFb/BMP signaling
ID3	TGFb/BMP signaling
ID4	TGFb/BMP signaling
IHH	Hedgehog signaling
INHA	TGFb/BMP signaling
INHBA	TGFb/BMP signaling
INHBB	TGFb/BMP signaling
ITGA5	Integrins
ITGA6	Integrins
ITGB1	Mesenchymal
ITGB5	Integrins
JAG1	Notch signaling
KIT	Oocyte
KITLG	Granulosa
LGR5	WNT signalling
LHCGR	Theca interna/steroidogenesis
LHX9	Oocyte
LTBP1	TGFb/BMP signaling
MCAM	Endothelial cell
MMP15	Extracellular matrix
MYH11	Smooth muscle cell
NCAM1	Epithelial–mesenchymal transition
NGFR	Mesenchymal
NOTCH1	Notch signaling
NOTCH2	Notch signaling
NOTCH3	Notch signaling
NR5A1	Theca interna/steroidogenesis
NT5E	Mesenchymal
PECAM1	Endothelial cell
PGR	Steroidogenesis
POU5F1	Germ cell
PTCH1	Hedgehog signaling
PTCH2	Hedgehog signaling
SFRP2	WNT signalling
SNAI2	Epithelial–mesenchymal transition
STAR	Theca interna/steroidogenesis
SYCP3	Meiosis
TCF21	Stroma and Theca cells
TGFBR1	TGFb/BMP signaling
TGFBR3	TGFb/BMP signaling
THBD	Endothelial cell
THBS1	Granulosa
THY1	Mesenchymal
TOX3	Theca interna
TP63	Oocyte
VCAM1	Theca interna
WNT6	WNT signalling
WT1	Granulosa

(continued)

Supplementary Table S1. (continued)
