# Supplementary material for: Spatial transcriptomics in the human adult ovary: insights into key signalling pathways during follicular atresia
Source: Hum Reprod. 2026 Mar 26;41(6):929–39. doi: 10.1093/humrep/deag051 (PMC13230497; doi:10.1093/humrep/deag051)
Supplement: deag051_Supplementary_Table_S2 [file deag051_supplementary_table_s2.pdf]

Human Reproduction, 2026, 1–1
https://doi.org/10.1093/humrep/deag051
Supplementary Data

Supplementary Table S2. Donor, ovary, and follicle characteristics.

Follicle ID	ROI ID	Follicle type	Follicle diameter (mm)	Donor ID	Age donor (years)	Testosterone treatment	Duration treatment (days)	Phase ovary	Fibrosis
1	A1_3	type3_1	0.7	FTM120	18	Sustanon	1258	Follicular	No
2	A1_4	type2_1	1.9	FTM120					
3	A2_1	healthy_1	1.6	FTM32	21	Nebido	677	Follicular	No
4	A2_2	type2_2	2.9	FTM32					
5	A2_2	type1_1	1.3	FTM32					
6	A2_2	healthy_2	0.5	FTM32					
8	A2_2	healthy_7	0.9	FTM32					
9	A2_3	type2_3	2.9	FTM32					
10	A2_4	type3_2	1.0	FTM32					
11	B2_1	type1_2	1.8	FTM37	18	Sustanon	1184	Follicular	No
12	B2_1	type3_3	0.6	FTM37					
13	B2_3	healthy_3	0.5	FTM37					
14	B2_4	type3_4	0.9	FTM37					
15	C1_1	healthy_4	0.7	FTM31	26	Sustanon	1083	Follicular	No
16	C2_1	type1_3	0.6	FTM36	21	Sustanon	1109	Follicular	No
17	C2_2	type1_4	4.0	FTM36					
18	D1_1	type2_4	2.3	FTM31	26	Sustanon	1083	Follicular	No
19	D1_2	healthy_5	2.9	FTM31					
20	D1_3	type3_5	0.6	FTM31					
21	D2_1	healthy_6	1.9	FTM44	24	Nebido	1263	Follicular	No

© The Author(s) 2026. Published by Oxford University Press on behalf of European Society of Human Reproduction and Embryology.
This is an Open Access article distributed under the terms of the Creative Commons Attribution License (https://creativecommons.org/licenses/by/4.0/), which permits unrestricted reuse, distribution, and reproduction in any medium, provided the original work is properly cited.
Human Reproduction, 2026, 00, 1–16
https://doi.org/10.1093/humrep/deag051
Original Article
